# Exploring the perception of safety culture among nurses in Saudi Arabia

**DOI:** 10.1186/s12912-024-02077-7

**Published:** 2024-06-19

**Authors:** Essa H. Al Muharraq, Farida Abdali, Abeer Alfozan, Sultan Alallah, Bashaer Sayed, Abdulrahman Makakam

**Affiliations:** 1 Nursing Administration, , Al Darb General Hospital, Jazan, Saudi Arabia; 2 Nursing Administration, King Fahad Armed Forces Hospital, Jeddah, Saudi Arabia; 3 Pharmacy, Prince Mohammed Bin Nasser Hospital, Jazan, Saudi Arabia; 4 Nursing Administration, , Prince Mohammed Bin Nasser Hospital, Jazan, Saudi Arabia; 5 Nursing Administration, Eradah & Psychiatry Hospital, Jazan, Saudi Arabia

**Keywords:** Patient safety, Patient safety culture, Nursing, Hospitals, Saudi Arabia

## Abstract

**Background:**

Medical errors and adverse events pose a serious challenge to the global healthcare industry. Nurses are at the frontline in implementing safety measures and protecting patients. This study aimed to investigate nurses’ perceptions of the patient safety culture in Saudi Arabia.

**Methods:**

This cross-sectional descriptive study used convenience sampling to survey 402 nurses from various hospitals in Jazan, Saudi Arabia. The Hospital Survey on Patient Safety Culture was used for the data collection.

**Results:**

Nurses reported a moderate perception of safety culture, with 60% positive responses. Teamwork had the highest safety culture rating at 77.8%, while responses to error and staffing were the lowest at 39.75% and 46.17%, respectively. Qualifications significantly predicts nurses’ safety culture rating (B = -0442, t = -4.279, *p* < 0.01). Positive correlations were found between event reporting frequency and communication openness (*r* = 0.142, *p* < 0.01), and patient safety grades with communication about errors (*r* = 0.424, *p* < 0.01) and hospital management support (*r* = 0.231, *p* < 0.01).

**Conclusions:**

Nurses in Saudi Arabia demonstrated a strong sense of teamwork and commitment to organizational learning. However, critical areas such as staffing and error response require attention to improve patient safety.

## Background

Medical errors and adverse events pose a significant challenge to public health globally [1]. Roughly 1 out of 10 patients suffers harm in healthcare settings, contributing to an annual toll of > 3 million deaths caused by unsafe care practices. Notably, over 50% of these instances of harm are considered preventable [2]. This increase in such incidences highlights the global healthcare industry’s imperative to improve the patient safety culture. The groundbreaking report of the Institute of Medicine, “To Err Is Human,” elucidated the prevalence of medical errors in the United States, estimating that tens of thousands of preventable deaths occur annually. This report sparked a global initiative to prioritize patient safety as a key issue in healthcare [3, 4].

The concept of safety culture in healthcare has gained considerable attention in recent years, with various studies revealing its efficacy in improving patient outcomes and reducing adverse events [5, 6]. The Agency for Healthcare Research and Quality (AHRQ) defined the safety culture of an organization as the product of individual and group values, attitudes, perceptions, competencies, and behavior patterns that identify the commitment to and the style and proficiency of an organization’s health and safety management [7]. According to the World Health Organization, the implementation of safety culture interventions significantly reduces medical errors, decreases staff turnover, and improves morale [8]. Additionally, a positive safety culture helped develop a more satisfied and committed workforce, which may translate into better patient outcomes [9].

Nurses in healthcare organizations are instrumental in delivering high-quality patient care, frequently acting as the first line of defense to ensure patient safety [10]. Their pivotal role includes the prevention of medical errors and adverse events, which is crucial considering the complexity and demanding nature of their work. The high-stress environment endemic to nursing exacerbates the risk of errors, making them particularly vulnerable to mistakes and adverse events, potentially compromising the well-being of patients [11]. Several studies, such as those by Chegini et al. [12], have revealed a strong correlation between positive safety culture promotion and medical error reduction within nursing. A positive safety culture encourages open communication, organizational commitment to learning from mistakes, and unwavering patient safety prioritization [13]. Nurses in such environments are empowered to report errors without fear of retribution, and are actively involved in initiatives aimed at improving safety protocols. Workload and staffing levels play a significant role in nurses’ ability to provide high-quality patient care and safely manage complex and critical situations [14, 15]. Meanwhile, effective leadership, clear communication, and teamwork among nurses helped overcome many of the barriers that hindered a positive safety culture [16]. Introducing measures to improve safety culture has substantially reduced mortality rates, lowered staff attrition rates, and boosted overall morale [17–19].

The number of complaints and claims against healthcare providers due to mortality or morbidities associated with adverse events is increasing in the Saudi Arabian healthcare system [20, 21]. Ineffective leadership, blame culture, workload/inadequate staffing, and poor communication are the main factors that hinder a positive patient safety culture in Saudi Arabia [16, 22]. Common attributes in Saudi healthcare facilities include supportive organizational attitudes toward learning/continuous improvement, good teamwork within units, and support from hospital management for patient safety. However, evidence indicates that healthcare professionals in Saudi Arabia lack the appropriate awareness of cultural safety concepts, and these concepts are inadequately implemented in the healthcare system. Rawas and Abou Hashish [23] revealed that the overall positive response rate of the predictors of patient safety culture in the Hospital Survey on Patient Safety Culture (HSOPSC) survey was 63.46%, warranting a comprehensive safety culture program to improve the safety culture perception among nurses in Saudi Arabia. Furthermore, Kaud [24] conducted a review of patient safety in the Saudi healthcare system and revealed a lack of understanding of patient safety concepts, a need for a systematic approach to patient safety, and a more positive patient safety culture is associated with a lower occurrence of sentinel events [25]. The same review indicated the importance of leadership in developing and implementing a patient-centered safety culture and the need for organizational support to achieve this goal.

Given the complexity and interdependence within healthcare settings as outlined, this study employs Systems Theory as its foundational framework to understand the multifaceted interactions that influence patient safety culture among nurses in Saudi Arabia. von Bertalanffy described what has since become known as General Systems Theory, the concept that systems cannot be reduced to a series of parts functioning in isolation, but that, in order to understand the whole, one must understand the interrelations between these parts [26]. Systems Theory, which views organizations as interconnected and dynamic entities, is particularly pertinent for identifying how elements such as leadership effectiveness, staffing adequacy, and communication impact the safety culture. By applying this theory, the study will analyze not just isolated issues but the broader systemic interactions within nursing contexts, aiming to pinpoint key leverage here systemic changes can significantly enhance overall safety culture and patient safety.

The perception of safety culture among nurses is crucial to patient care outcomes; however, a comprehensive understanding of how nurses experience and interpret this culture within healthcare organizations remains unclear. This study aims to fill this gap by investigating nurses’ perceptions and understanding of patient safety culture in nursing to analyze existing cultures and determine strategies to build or shape them. This is particularly pertinent in Saudi Arabia, where the healthcare sector is undergoing significant transformations to meet the ambitious health standards set forth by Saudi Vision 2030. By exploring the intricate interactions and factors within healthcare settings that influence nurses’ perceptions, such as leadership dynamics, communication systems, and policy frameworks, the study will provide valuable insights. These findings will help healthcare leaders and policymakers to design and implement culturally relevant reforms that enhance patient safety culture, contribute to the improvement of healthcare quality and safety, and support the nation’s goal of becoming a leader in healthcare innovation.

## Methods

### Design

This study used a cross-sectional descriptive design.

### Setting

Nurses from a range of departments within two major tertiary hospitals and five general hospitals in Jazan, Saudi Arabia, were recruited. These hospitals are known for their comprehensive healthcare services and are appropriate settings for investigating a wide spectrum of safety culture attributes.

### Participants and sampling

The study used convenience sampling and calculated the sample size using the Taro-Yamen formula for a finite population. Assuming a sample size of *n* = 338 yielded a significance level of α = 0.05. The study sample consisted of 402 nurses employed at two major tertiary and five general hospitals in Jazan, Saudi Arabia. The inclusion criterion was registered nurses who were currently employed by one of the hospitals. To maintain a focus on bedside care providers, nursing staff working in Outpatient Departments and who held managerial positions without direct patient care duties were excluded.

### Instrumentation

The HSOPSC, developed by AHRQ, was specifically designed to assess the patient safety culture of healthcare settings. The HSOPSC includes 32 items that evaluate different dimensions of the patient safety culture. The tool was used nationally in previous studies that assessed the perception of nurses on the patient safety culture in Saudi Arabia [27, 28].

The HSOPSC consists of 10 dimensions, namely: teamwork within units, supervisor expectations and actions promoting patient safety, organizational learning–continuous improvement, management support, feedback and communication about errors, communication openness, teamwork across units, staffing, handoffs and transitions, and non-punitive response to errors. The HSOPSC is a validated tool with Cronbach’s alpha ranging from 0.63 to 0.84, which suggests acceptable to good reliability. In order to verify the reliability and applicability of the questionnaire used in this study, a pilot study was conducted with the participation of 15 nurses. The results showed Cronbach’s α coefficient of 0.80.

Respondents rated each item on a five-point Likert scale, indicating the level of agreement or frequency on a scale ranging from 1 (strongly disagree) to 5 (strongly agree) or from 1 (never) to 5 (always). Additionally, the HSOPSC includes an overall patient safety culture rating, in which participants evaluate the level of patient safety in their specific work area or unit, ranging from “excellent” to “poor.”

### Procedure for data collection

The primary data of the study was collected between July and August 2023. The researchers collaborated with the regional nursing administration to organize meetings with nurses and personally handed out the survey forms to the participants. During these meetings, the researchers clarified the study’s objective and informed the nurses that their consent to participate would be assumed upon the submission of the completed survey. Every participant was provided with a sealable envelope to ensure their answers’ confidentiality and anonymity. Out of the 500 questionnaires that were handed out, 402 were returned, resulting in a response rate of 80.4%.

### Ethical considerations

This study obtained ethical approval from Jazan Health Affairs before the commencement of data collection. The survey began with an informed consent cover page, which all participants were required to read before completing the questionnaire. The participants were informed that submitting the completed survey would constitute their consent to participate in the study. Rigorous measures have been established to safeguard the privacy and confidentiality of participant data throughout the entire process.

### Statistical analysis

The Statistical Package for the Social Sciences version 25.0 was used. Descriptive statistics, such as means and percentages of responses, were used to summarize the HSOPSC survey data. Regression analysis was employed to assess the impact of staff demographics on their ratings of safety culture. Pearson correlation analysis was used to investigate the association between measures of patient safety culture and two key variables: the overall patient safety grade and the frequency of events reported.

## Results

**Table 1:** Demographic characteristics of participants

Demographic variables	Category	Frequency	Percentage
Gender	Male	64	15.9
	Female	338	84.1
Qualification	Diploma	138	34.3
	Bachelor	241	60.0
	Master	23	5.7
Nationality	Saudi	349	86.8
	Non-Saudi	53	13.2
Marital Status	Single	148	36.8
	Married	228	56.5
	Divorced	21	5.5
	Widowed	5	1.2
Age, year	18–30	220	54.7
	31–45	172	42.8
	> 46	10	2.5
Experience	0–10	295	73.4
	11–25	104	25.9
	> 25	3	0.7

The total sample size of participants was 402 nurses, consisting predominantly of female nurses (84.1%) with bachelor’s degrees (60.0%). The majority were Saudi nationals (86.8%) and were married (56.7%). Most nurses were young, aged 18–30 years (54.7%), and had 0–10 years of experience (73.4%); see (Table 1).

**Table 2:** Nurse perceptions of safety culture

Items		Strongly disagree/disagree	Neutral	Strongly agree/agree	Average positive response
**Teamwork**					77.8%
We work together as an effective team in this unit		6 (1.5%)	23 (5.7%)	346 (92.7%)	346 (92.7%)
Staff in this unit help each other during busy times,		10 (2.4%)	21 (5.2%)	370 (92%)	370 (92%)
Staff working in this unit have a problem with disrespectful behavior		196 (48.8%)	86 (21.4%)	84 (20.9%)	196 (48.8%)
**Staffing and Work Pace**					46.17%
We have enough staff to handle the workload in this unit		131 (32.6%)	57 (14.2%)	204 (50.7)	204 (50.7%)
Staff in this unit work longer hours than is best for patient care		55 (13.7%)	61 (15.2%)	273 (67.9%)	55 (13.7%)
This unit relies too much on temporary, float, or PRN staff		134 (33.3%)	93 (23.1%)	134 (33.3%)	134 (33.3%)
This unit has a fast working pace that negatively affects patient safety		183 (45.5%)	95 (23.6%)	101 (25.2%)	183 (45.5%)
**Organizational Learning—Continuous Improvement**					72.3%
This unit regularly reviews work processes to identify the necessity of changes to improve patient safety		31 (7.7%)	37 (9.2%)	327 (81.4%)	327 (81.4%)
In this unit, changes to improve patient safety are evaluated to identify their effectiveness		28 (7%)	32 (8%)	337 (83.8%)	337 (83.8%)
This unit allows the same patient safety problems to persist		208 (51.7%)	72 (17.9%)	90 (22.4%)	208 (51.7%)
**Response to Error**					39.75%
Staff in this unit believe that their mistakes are being used against them		135 (33.6%)	100 (24.9%)	141 (35%)	135 (33.6%)
Incidence reported in this unit seemed to document the person and not the problem		126 (31.3%)	89 (22.1%)	168 (41.8%)	126 (31.3%)
This unit focuses on learning rather than blaming individuals during errors		67 (16.7%)	69 (17.2%)	254 (63.2%)	254 (63.2%)
Support for staff involved in patient safety errors is lacking in this unit		124 (30.9%)	105 (26.1%)	139 (34.6%)	124 (30.9%)
**Supervisor, Manager, or Clinical Leader Support for Patient Safety**					59.56%
My supervisor, manager, or clinical leader seriously considers staff suggestions for improving patient safety		39 (9.7%)	49 (12.2%)	300 (74.6%)	300 (74.6%)
My supervisor, manager, or clinical leader wants us to work faster during busy times, even if it indicates taking shortcuts		108 (26.8%)	92 (22.9%)	182 (45.3%)	108 (26.8%)
My supervisor, manager, or clinical leader takes action to address patient safety concerns that are raised to their attention		24 (6%)	54 (13.4%)	311 (77.3%)	311 (77.3%)
**Communication About Error**					69.36%
We are informed about errors that happen in this unit		39 (9.7%)	84 (20.9%)	275 (68.4%)	275 (68.4%)
We discuss ways to prevent errors from persisting in this unit		44 (11%)	56 (13.9%)	295 (73.4%)	295 (73.4%)
We are informed about changes that are made based on event reports in this unit		37 (9.2%)	82 (20.4%)	267 (66.3%)	267 (66.3%)
**Communication Openness**					55.75%
Staff speak up about things that may negatively affect patient care in this unit		33 (8.2%)	73 (18.2%)	289 (71.9%)	289 (71.9%)
Staff in this unit speak up about someone with more authority doing something unsafe for patients		79 (19.6%)	67 (16.7%)	241 (60%)	241 (60%)
Those with more authority are open to their patient safety concern when staff speak up in this unit		52 (12.9%)	100 (24.9%)	210 (52.3%)	210 (52.3%)
Staff in this unit are afraid to ask questions when something does not seem right		156 (38.8)	118(29.4%)	108 (26.9%)	156 (38.8)
**Reporting Patient Safety Events Dimension**					59.45%
How often is it reported when a mistake is caught and corrected before reaching the patient		63(15.7%)	78(19.4%)	238(59.2%)	238(59.2%)
How often is it reported when a mistake reaches the patient and could have harmed the patient, but did not		67(16.7%)	69(17.2%)	240(59.7%)	240(59.7%)
**Hospital Management Support for Patient Safety dimension.**					61.1%
The actions of hospital management indicate that patient safety is a top priority		17(4.2%)	37(9.2%)	342(85.1%)	342(85.1%)
Hospital management provides adequate resources to improve patient safety		40(9.9%)	72(17.9%)	280(69.6%)	280(69.6%)
Hospital management seems interested in patient safety only after an adverse event happens		115(28.6%)	97(24.1%)	163(40.6%)	115(28.6%)
**Handoffs and Information Exchange dimension.**					58.3%
Important information is frequently left out when transferring patients from one unit to another		199(49.5%)	85(21.1%)	88(21.9%)	199(49.5%)
Important patient care information is frequently left out during shift changes		209(52%)	83(20.6%)	91(22.7%)	209(52%)
Time to exchange all key patient care information is adequate during shift changes		36(8.9%)	61(15.2%)	295(73.4%)	295(73.4%)
**Nurses’ Patient Safety Ratings**	Poor	Fair	Good	Very Good	Excellent
	7(1.7%)	46(11.5%)	105 (26.1%)	109 (27.1%)	135 (33.6%)

Teamwork was the most highly rated dimension, with an average positive response of 77.8%, followed by organizational learning (with a 72.3% positive response) and communication about error (69.36% positive response). In contrast, response to error scored the lowest positive score among all dimensions (39.75%), followed by staffing levels, which received the second lowest average positive rating at 46.17%, (Table 2).

**Table 3:** Regression Analysis of Nurses’ Ratings for Safety Culture Based on Demographic Characteristics

	*R*	*R* square	Adjusted *R* square	Std. error	
	0.242	0.059	0.044	1.059	
	Sum of squares	Df	Mean square	F	*p*
Regression	27.497	6	4.583	4.088	0.001
Residual	441.734	395	1.121		
Total	469.232	401			
	Unstandardized coefficients (B)	Std. error	Standardized coefficients (B)	T	*p*
Constant	5.224	0.529		9.868	0.001
Age	-0.009	0.015	-0.054	-0.650	0.516
Gender	-0.052	0.154	-0.018	-0.339	0.735
Marital status	0.018	0.090	0.011	0.204	0.838
Nationality	-0.238	0.174	-0.074	-1.367	0.172
Experience as a nurse	-0.006	0.016	-0.033	-0.384	0.701
Qualification	-0.442	0.103	-0.230	-4.279	0.001

The regression analysis exploring the impact of demographic factors on nurses’ ratings for safety culture demonstrates that while the model is statistically significant, it explains only a minor portion of the variance (5.9%). Among the demographic variables assessed only ‘Qualification’ showed a significant (B = -0442, t = -4.279, *p* < 0.001), see Table 3.

**Table 4:** Correlation between patient safety culture measures on overall patient safety grade and frequency events reported

Items		Teamwork	Staffing and work pace	Organizational learning—continuous improvement	Response to error	Supervisor, manager, or clinical leader support	Communication about error	Communication openness	Reporting patient safety events	Hospital management support for patient safety	Handoffs and information exchange
Number of events reported	*r*	0.018	0.002	0.069	0.048	0.033	0.024	0.142^**^	0.182^**^	0.014	0.081
	*P*	0.724	0.969	0.170	0.339	0.504	0.628	0.004	0.001	0.776	0.105
Patient Safety Rating	*r*	0.104^*^	0.020	0.190^**^	−0.223^**^	0.210^**^	0.424^**^	0.081	0.186^**^	0.231^**^	−0.055
	*P*	0.037	0.693	0.001	0.001	0.001	0.001	0.104	0.001	0.001	0.272

Positive correlations were found between the frequency of event reporting and communication openness (*r* = 0.142, *p* < 0.01), and nurses’ reports of patient safety events (*r* = 0.182, *p* < 0.01). Patient safety grades were correlated with communication about error (*r* = 0.424, *p* < 0.01), hospital management support (*r* = 0.231, *p* < 0.01), clinical leader support (*r* = 0.210, *p* < 0.01), organizational learning and continuous improvement (*r* = 0.190, *p* < 0.01), teamwork (*r* = 0.104, *p* < 0.05), and response to error (*r* = − 0.223, *p* < 0.01) (Table 4).

## Discussion

The patient safety culture is crucial in healthcare settings. This study aimed to explore perceptions of safety culture among nurses in Saudi Arabia. In this study, nurses reported a moderate level of safety culture perception, with a 60% positive response. However, there is significant variance across specific dimensions that warrant closer examination. The teamwork dimension recorded the highest positive response rate (77%), which is similar to the average rate of AHRQ (78%) and higher than that in studies conducted in Iran (65%) [12] and South Korea (44%) [13]. The results confirmed that the healthcare field relies heavily on the collaborative work of diverse groups of specialists [29].

However, only 46.17% of the respondents provided positive feedback concerning staffing levels. This finding aligns with global challenges in nursing shortages and retention, highlighting a significant issue in Saudi Arabia’s healthcare system as well. Previous studies that have proposed an association between nursing staffing levels and safety culture support this finding suggesting that insufficient staffing not only increases stress and job dissatisfaction but also heightens the risk of burnout and compromises patient care quality [1, 30, 31]. This is also consistent with Upadhyay et al. (2021), who indicated an indirect effect of registered nurse staffing on safety culture perceptions [32]. To address this, healthcare facilities should consider strategies such as improving nurse-to-patient ratios, implementing flexible scheduling to reduce burnout, and using part-time or floating staff during peak periods. These measures can alleviate staff workload, potentially decreasing job dissatisfaction and enhancing patient care quality. A positive response to the organizational learning—continuous improvement dimension indicated that most nurses felt that their units were committed to improving patient safety through ongoing process of reviews and enhancements. This result is supported by Abuosi et al. [1] and Rawas and Hashish [23] in which high positive score indicates the presence of dynamic work culture. Such environments are characterized by continuous evaluation, learning, and adaptation, which are essential elements for maintaining and enhancing safety standards.

The response to errors within nursing units was identified as a critical area requiring improvement, as evidenced by the lowest positive response rates among the survey domains. The results indicated a notable disconnect between current error management practices and the principles of a non-punitive approach that is essential for patient safety. The blame culture could discourage the transparent reporting of errors and hinder the potential for systemic learning and improvement, which is well documented to be crucial for patient safety [33, 34]. Remarkably, the communication about error dimension showed a positive response rate, indicating that a majority of nursing staff felt adequately informed about errors and the subsequent preventive actions taken within their unit. This represents a proactive approach to error communication and commitment to continuous improvement, which are key factors in promoting a strong safety culture [35, 36]. The reluctance to report errors due to fear of blame is a significant barrier to improving safety culture. Hospitals should implement and enforce non-punitive error reporting policies. Creating an environment where errors are openly discussed and viewed as opportunities for learning rather than occasions for punishment could significantly enhance error reporting rates. The findings for communication openness in the study yielded a positive response rate of 55.75%, indicating a foundation for open communication within units, with significant room for improvement. This result is consistent with other studies in which the level of communication openness about errors was equipollent among participants [13, 37]. Improving open communication is crucial for maintaining patient safety, as it encourages reporting and collaborative problem-solving. Healthcare facilities should implement regular, structured communication forums such as briefings and debriefings, which can help ensure all team members are aware of ongoing issues and the measures being taken to address them.

Leadership also plays a crucial role; a positive perception of leadership supports patient safety among nursing staff. Hospital management and nurse managers should demonstrate inclusiveness by seeking opposing viewpoints, demonstrating a willingness to listen and respond to staff concerns and recommendations, show appreciation for staff input, and treat the staff with respect. The positive notion of participants about management commitment to safety is consistent with other studies conducted in Saudi Arabia [3, 38]. Therefore, healthcare leaders should undergo training that emphasizes open communication, transparency, and inclusiveness. Leadership programs should also focus on recognizing and appreciating staff efforts, boosting morale and promoting a proactive safety culture. A small majority of the staff reported patient safety events. This rate reveals the level of engagement in reporting safety events, especially when errors are intercepted before reaching the patient or when an error reaches the patient but causes no harm. The positive response rate indicates that healthcare staff recognize the importance of reporting both near misses and no-harm events, which is essential for learning and improving patient safety systems [39]. Effective handoffs and clear information exchanges are crucial for ensuring patient safety and continuity of care, as they minimize the risk of information loss and misunderstandings that could lead to errors [40]. This gap may be due to various factors such as inadequate communication systems, insufficient time allocated for handoffs, lack of standardized procedures, or limited training on effective communication strategies [35].

The regression analysis revealed that higher qualifications among nurses predict lower safety culture ratings. Nurses with higher qualifications typically have access to more extensive education and training, which likely increases their exposure to best practices and ideal safety standards. This enhanced exposure can elevate their expectations for their work environments, leading them to assess their workplace safety culture more critically when these expectations are not met. Additionally, advanced education may equip nurses with a sharper ability to identify and understand systemic flaws and safety lapses that might be overlooked by others [41]. This study identified significant correlations between dimensions of safety culture and both the perceptions of overall safety and the frequency of event reporting, aligning with findings by Abuosi et al. [1] and Saleh et al. [40]. Notably, open communication and a non-punitive environment correlated weakly but positively with increased event reporting. This suggests that a culture where nurses feel comfortable speaking, asking questions, and discussing errors openly might contribute to a slight increase in the reporting of safety events [42]. However, the weak nature of this correlation indicates that other factors might also play significant roles in influencing event reporting. Therefore, while fostering open communication and a non-punitive culture is beneficial, these measures alone may not be sufficient to significantly enhance event reporting rates. Similarly, Falcone et al. [43] highlighted the positive effect of promoting open communication and non-punitive responses to error reporting on the rates at which events are reported.

Teamwork, leadership support, organizational learning and communication about errors exhibited only weak to moderate positive correlations with higher patient safety grades, despite their recognized importance in previous studies [3, 39, 44]. These findings, which emphasize the role of collaborative environments and ongoing improvement efforts in achieving better safety outcomes, should be interpreted with caution due to the mild strength of the correlations. Conversely, a blame culture was negatively correlated with safety perceptions, highlighting the need for healthcare settings to shift toward more supportive, learning-focused approaches [45, 46].

The study offers valuable insights. However, its use of self-reported data from a non-representative sample of nurses in a single region of Saudi Arabia presents certain generalizability limitations. The cross-sectional design restricts the ability to ascertain causality or observe trends over time. Moreover, the lack of objective criteria and the presence of potential confounding variables, such as different hospital regulations and differing degrees of staff training, may mask the actual impact of safety culture on patient outcomes. To address existing limitations and enhance the rigor of future research, it is recommended to broaden the demographic and geographic scope of the participant base, and integrate a mix of qualitative and quantitative data. This approach will provide a more comprehensive understanding of the dynamics influencing safety culture within healthcare environments.

## Conclusion

The current study on patient safety culture among nurses in Saudi Arabia revealed a strong sense of teamwork and a positive approach to organizational learning. However, it also emphasizes significant concerns about staffing which warrants resolution to improve patient safety. The study indicates that communication about errors and response to errors require improvement despite a generally favorable view of leadership support. Overall, the results indicate both commendable strengths and critical areas for development to foster a culture that ensures patient safety and care.

## Data Availability

The datasets analyzed during the current study are available from the corresponding author on reasonable request.

## Abbreviations

AHRQ: Agency for Healthcare Research and Quality
HSOPSC: Hospital Survey on Patient Safety Culture
WHO: World Health Organization
